# Ovariectomy results in differential shifts in gut microbiota in low versus high aerobic capacity rats

**DOI:** 10.14814/phy2.12488

**Published:** 2015-08-11

**Authors:** Kimberly A Cox-York, Amy M Sheflin, Michelle T Foster, Christopher L Gentile, Amber Kahl, Lauren G Koch, Steven L Britton, Tiffany L Weir

**Affiliations:** 1 Department of Food Science and Human Nutrition, Colorado State UniversityFort Collins, Colorado, USA; 2 Department of Anesthesiology, University of Michigan Medical SchoolAnn Arbor, Michigan, USA

**Keywords:** Aerobic capacity, gut microbiota, menopause

## Abstract

The increased risk for cardiometabolic disease with the onset of menopause is widely studied and likely precipitated by the decline in endogenous estradiol (E_2_), yet the precise mechanisms are unknown. The gut microbiome is involved in estrogen metabolism and has been linked to metabolic disease, suggesting its potential involvement in the postmenopausal phenotype. Furthermore, menopause-associated risk factors, as well as gut ecology, are altered with exercise. Therefore, we studied microbial changes in an ovariectomized (OVX vs. Sham) rat model of high (HCR) and low (LCR) intrinsic aerobic capacity (*n *=* *8–10/group) in relation to changes in body weight/composition, glucose tolerance, and liver triglycerides (TG). Nine weeks after OVX, HCR rats were moderately protected against regional adipose tissue gain and liver TG accumulation (*P *<* *0.05 for both). Microbial diversity and number of the Bacteroidetes phylum were significantly increased in LCR with OVX, but unchanged in HCR OVX relative to Sham. Plasma short-chain fatty acids (SCFA), produced by bacteria in the gut and recognized as metabolic signaling molecules, were significantly greater in HCR Sham relative to LCR Sham rats (*P *=* *0.05) and were decreased with OVX in both groups. These results suggest that increased aerobic capacity may be protective against menopause-associated cardiometabolic risk and that gut ecology, and production of signaling molecules such as SCFA, may contribute to the mediation.

## Introduction

The menopause transition is characterized by an increased risk for cardiometabolic disease (e.g., cardiovascular disease, type 2 diabetes, and fatty liver) concurrent with decreased energy expenditure and increases in total adiposity. The loss of endogenous hormones (17-*β* estradiol; E_2_) has been implicated, as E_2_ replacement in human and animal models of menopause has been shown to prevent or reverse many of the adverse health effects associated with menopause (Salpeter et al. 2006). The precise mechanisms, however, are unresolved.

One potential mechanism is an estrogen-mediated change in the gut microbial community. Strong correlations have been made between the composition of gut microbes and development of metabolic disease, in both human and animal models (Joyner et al. 2000; Larsen et al. 2010; Howitt and Garrett 2012; Zhang et al. 2013). Moreover, sex hormones (e.g., estrogen and testosterone) have been shown to influence microbial communities (Kornman and Loesche 1982; Markle et al. 2013), and gut microbes reciprocally affect the metabolism and systemic levels of these hormones (Adlercreutz et al. 1984; Plottel and Blaser 2011; Flores et al. 2012). For estrogen, these effects appear to be mainly regulated by estrogen receptor beta (ER*β*), which is the primary estrogen receptor in the gut. Intestinal ER*β* status affects the composition of microbiota in female mice (wild type vs. ER*β* knockout), and is further modulated by diet (Menon et al. 2013). ER*β* also appears to have a major role in intestinal health as epithelial ER*β* mRNA is upregulated in patients with Crohn’s disease and ulcerative colitis (van Looijer-Langen et al. 2011); diseases associated with inflammatory microbial profiles and increased gut permeability (Gevers et al. 2014).

Exercise and “physical fitness” (defined by VO2max/anaerobic threshold) have been shown to slow the progression and mitigate the degree of menopause-associated metabolic disturbances (Sternfeld and Dugan 2011). In menopausal women and ovariectomized (OVX) rats, exercise training mitigates increases in visceral adipose tissue, liver triacylglycerol, and plasma insulin relative to untrained subjects (Pighon et al. 2011). Some of the effect is due to increased energy expenditure and weight maintenance; however, exercise training restores the expression of many of the genes involved in adipogenesis and lipid accumulation in liver that are altered with OVX to levels similar to those seen with E_2_ treatment (Pighon et al. 2011). Therefore, exercise produces estrogen-like effects independent of effects on body weight and adiposity.

It is possible that some of these effects are driven by the microbial community of the gut. Five weeks of voluntary exercise (free access to running wheel) in rats and mice significantly altered intestinal microbiota relative to sedentary controls (Matsumoto et al. 2008; Choi et al. 2013). A similar study is currently underway to investigate the effects of diet and exercise on the gut microbiome in postmenopausal women and men (Liu et al. 2014). The mechanisms driving exercise-mediated regulation of the gut microbial population are not known, however, they may include regulation of primary and secondary bile acids (Hagio et al. 2010; Meissner et al. 2011), short-chain fatty acids (Matsumoto et al. 2008), and components of the immune system (Viloria et al. 2011).

The aim of the current study was to investigate the effects of inherent aerobic capacity and loss of endogenous estrogen (OVX) on the microbial colonization of the gut. While prescribed, moderate to intense exercise is the standard in controlled trials, there are acute effects of individual bouts of exercise on metabolic outcomes (e.g., plasma lipids, glucose, insulin, and energy consumption) (Herd et al. 1998; Burton et al. 2008), making it difficult to separate innate aerobic capacity from exercise conditioning. We therefore chose low (LCR) and high (HCR) running capacity rats developed by Koch and Britton (Britton and Koch 2001; Koch and Britton 2001; Wisloff et al. 2005) to model these effects. The LCR/HCR rats are derived from several rat strains and are bred based on their running capacity (time and distance of treadmill running). In an experimental setting, innate aerobic capacity is on the backdrop of caged-activity, which limits the effect of a single bout of prescribed exercise performed just prior to endpoint measures. Moreover, the genetic heterogeneity of the rats may be considered more representative of the polygenic human metabolic condition than a select pure strain. We hypothesized that OVX-induced changes in body weight and adiposity would be mitigated by increased innate aerobic capacity, and associated with changes in the composition of the gut microbiota.

We demonstrate that greater innate aerobic capacity (HCR) modestly mitigated OVX-induced AT and liver TG accumulation in this population of rats. We also observed a significant change in microbial diversity in the LCR-OVX group, relative to LCR-SHAM, but not in the respective HCR groups. These results indicate that endogenous ovarian hormones influence gut ecology, and that increased aerobic capacity may prevent or delay menopause-associated lipid accumulation.

## Methods

### Animals and experimental design

Animal care and procedures were approved by the Colorado State University Institutional Animal Care and Use Committee, and all conditions meet or exceed standards as described in the Animal Welfare Act regulations and the Guide for the Care and Use of Laboratory Animals.

Female rats bred for low and high aerobic capacity (LCR and HCR, respectively) were obtained from the University of Michigan (Ann Arbor, MI). Rats were derived from a founder population of 192 genetically heterogeneous rats (96 male and 96 female; NIH stock), as previously described (Koch and Britton 2001). Upon arrival, rats (32 weeks of age) were housed individually in a temperature- and humidity-controlled environment with 12 h light:dark cycle and provided a standardized low-fat diet (Teklad diet 2918, Harlan) and water ad libitum. Two weeks prior to surgery, rats were switched to a phytoestrogen-free diet (Teklad 2020X Harlan; 24% PRO, 16% FAT, 60% CHO) ad libitum. At 35 weeks of age rats underwent sham (Sham, midline incision through skin and muscle) or ovariectomy (OVX, midline incision followed by bilateral ligation of fallopian tubes and resection of ovaries). Muscle was sutured together and skin was closed with wound clips. Body weight and food intake were measured weekly for 9 weeks. Groups were: LCR Sham (*n *=* *9), LCR OVX (*n *=* *10), HCR Sham (*n *=* *11), and HCR OVX (*n *=* *8).

### Ip gtt

Glucose tolerance tests (GTTs) were conducted 2 weeks before experiment termination. Rats were fasted for 4 h. Blood glucose was then determined from tail vein blood (Freestyle Lite Glucometer; Abbott, Abbott Park, IL), before intraperitoneal injection of 1.5 g/kg dextrose. Blood glucose was again assessed from tail vein blood samples at 15, 30, 45, 60, and 120 min postinjection.

### Tissue collection

Rats were fasted 4 h prior to termination. Animals were anesthetized with isoflurane and euthanized via exsanguination. Blood was collected into EDTA-coated tubes, centrifuged, and plasma was stored at −80°C for later analysis. Liver and adipose tissue (AT) were removed and frozen in liquid nitrogen. Tissues were stored at −80°C until further analysis. Adipose tissue depots and uterus were weighed before freezing.

### Fasting glucose

Animals were fasted for 4 h before termination. Blood was obtained via cardiac puncture into EDTA-coated tubes and centrifuged at 3500 *g* for 15 min to obtain plasma. Plasma was analyzed for glucose via Sigma Glucose Assay Reagent (St. Louis, MO) as per manufacturer’s instructions.

### Liver triglyceride levels

Liver tissue was weighed and digested in ethanolic potassium hydroxide. Samples then underwent multiple ethanol solvent purification steps and were precipitated via magnesium chloride. The supernatant was removed and triglyceride content was assayed using the Cayman Triglyceride Colorimetric Assay Kit (Ann Arbor, MI) as per manufacturer’s instructions.

### Characterization of the fecal microbiota

Fecal samples were collected from four animals from each group (total *n* = 16) for amplification and pyrosequencing of the V3–V5 region of the 16s rRNA gene. DNA was extracted from all samples using a MoBio Powersoil DNA extraction kit (MoBio, Carlsbad, CA) according to the manufacturer’s instructions. All samples were quantified with the Quanti-iT PicoGreen dsDNA assay (Life Technologies, Grand Island, NY) and stored at −20°C prior to shipping for sequence analysis. PCR library preparation and pyrosequencing was performed under contract with Research and Testing Laboratory (Lubbock, TX) using a 454 Life Sciences GS FLX System with titanium chemistry.

Multivariate statistical analysis was conducted using the adonis function of the vegan package (Oksanen et al. 2015) using R. The adonis function is a nonparametric method analogous to analysis of variance (ANOVA) and was utilized to determine differences in overall bacterial community composition across treatment groups. Adonis was used rather than the alternative analysis of similarities (Clarke 1993) because adonis can accommodate both continuous and categorical predictors and their interactions (Oksanen et al. 2015). *P*-values were based on 999 permutations. Graphical visualization of differences in fecal bacterial taxonomy between OVX and Sham HCR/LCR rats was accomplished via principal coordinate analysis using the Jaccard distance matrix output from MOTHUR and plotted in R (sup. 1). In addition to multivariate testing with adonis, the mother implementation of molecular analysis of variance (AMOVA) was used to confirm adonis tests. Specific pairwise tests were performed to detect differences in bacterial communities with OVX as compared to SHAM (i.e., HCR OVX was compared to HCR SHAM and LCR OVX was compared to LCR SHAM). To determine which bacterial OTUs showed significantly altered abundance with OVX compared to SHAM, metastats analysis was performed separately for LCR and HCR samples (http://metastats.cbcb.umd.edu/). The metastats analysis was also performed to determine which bacterial OTUs differed in abundance in HCR versus LCR (no OVX) samples. Bacterial diversity indices and relative abundance of phyla were compared via the nonparametric Kruskal–Wallis one-way analysis of variance by ranks test using R 3.1.1 (www.r-project.org). For all experiments, differences among groups were considered statistically significant if *P *≤* *0.05.

### Gut permeability

#### FITC dextran

At the experiment mid-point (5 weeks), intestinal permeability was assessed in vivo. Following a 6-h fast, rats were orally gavaged with 40 kD FITC-Dextran (Sigma, St. Louis, MO) dissolved in deionized water at 400 mg/kg body weight. After 1 h, ∼200 *μ*L of blood was collected via tail vein. Blood was then centrifuged at 12 000 *g* at 4°C for 3 min to separate plasma. Plasma was diluted in an equal volume of PBS, pH 7.4, and fluorescence read at 485_EX_/535_EM_. Concentration was calculated based on a standard curve produced from serial dilution of nontreated, plasma diluted 1:2 with PBS and spiked with FITC-dextran (0–10 *μ*g/mL).

#### Plasma endotoxin

Plasma endotoxin (ET) was determined using ToxinSensor L00350 (GenScript, Piscataway, NJ) as per manufacturer’s protocol. All steps were carried out in ET-free glass and plasticware using ET-free water and reagents. Briefly, following 1:20 dilution of plasma samples in water and heating at 70°C for 10 min, standards and samples were mixed with the supplied chromogenic substrate (Limulus Amebocyte Lysate) and incubated for 45 min at 37°C. Color stabilizing reagents were then added in sequence and 200 *μ*L was transferred to a 96-well plate to read A_545._ Baseline absorbance (A_545_) of diluted plasma before addition of the chromogenic substrate was subtracted from final results, and concentration was calculated from the standard curve and adjusted for the total dilution factor.

### Fecal short-chain fatty acids (SCFA)

Stool samples were extracted for short-chain fatty acids by mixing weighed frozen feces with acidified water (pH 2.5) containing 1 mmol/L of ethylbutyric acid as an internal standard and sonicated for 10 min. Samples were centrifuged and filtered through 0.45 *μ*mol/L nylon filters and stored at −80°C prior to analysis. The samples were analyzed using an Agilent 6890 Series Gas Chromatographer (Agilent Inc, Santa Clara, CA). Samples were injected at a 10:1 split ratio, and the inlet was held at 22°C and transfer line was held at 230°C. Separation was achieved on a 30 m TG-WAX-A column (Thermo Scientific, 0.25 mm ID, 0.25 *μ*m film thickness) using a temperature program of 100°C for 1 min, ramped at 8°C/min to 180°C, held at 180°C for 1 min, ramped to 200°C at 20°C/min, and held at 200°C for 5 min. Helium carrier flow was held at 1.2 mL/min. SCFA’s were quantified using standards of commercially purchased compounds and samples were adjusted for extraction efficiency differences by normalizing to the internal standard.

### Statistical analysis

Animal data were analyzed using one-way analysis of variance (ANOVA) (IBM SPSS for Windows, release 21; SPSS, Chicago, IL) with Tukey post hoc analyses. Bacterial diversity measurements were compared via ANOVA using R 3.0.1 (www.r-project.org). Multivariate ANOVA based on dissimilarities (Anderson 2001) was conducted on relative abundance of fecal microbiota to explore variation in composition associated with OVX or HCR/LCR status. A traditional Jaccard distance matrix, which considers both total and shared OTUs, was created in MOTHUR. Genetic distance of 7% was utilized to most closely approximate phylum level differences. Multivariate statistical analysis was conducted using the adonis function of the vegan package (Oksanen et al. 2015) using R. The adonis function is a nonparametric method analogous to analysis of variance (ANOVA) and was utilized to determine differences in bacterial diversity as well as taxonomy across treatment groups. Adonis was used rather than the alternative analysis of similarities (Clarke 1993) because adonis can accommodate both continuous and categorical predictors and their interactions (Oksanen et al. 2015). *P*-values were based on 999 permutations. Graphical visualization of differences in fecal bacterial taxonomy between OVX and Sham HCR/LCR rats was accomplished via principal coordinate analysis (PCoA) using the Jaccard distance matrix output from MOTHUR and plotted in R. In addition to multivariate testing with adonis, the mother implementation of molecular analysis of variance (AMOVA) was used to perform specific pairwise tests between treatment groups for bacterial taxonomy analysis. The MOTHUR implementation of metastats at 7% genetic distance was used to identify OTUs that were significantly different in abundance across treatment groups. For all experiments, differences among groups were considered statistically significant if *P *≤* *0.05.

## Results

### HCR rats are moderately protected from OVX-induced metabolic derangement

#### Body weight, food intake, tissue weights

**Figure 1:**
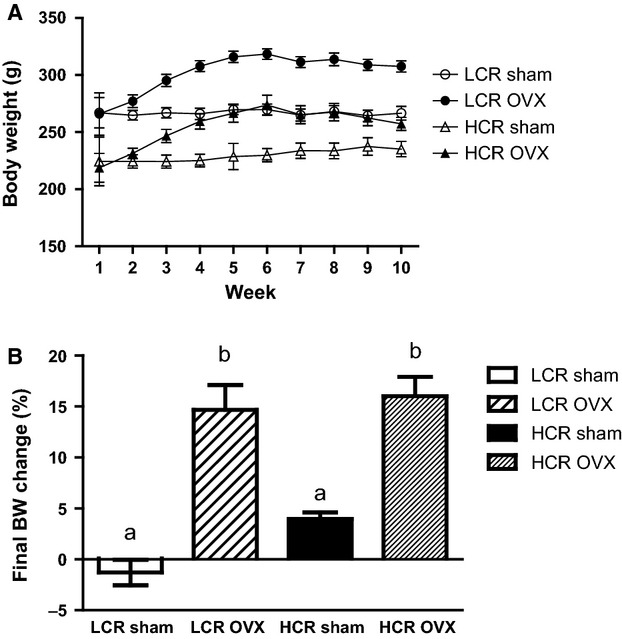
HCR rats weigh significantly less than LCR across treatments, but gain equivalent percent body mass with OVX. Weekly body weight gain (A) and percent total body weight gain (B) over 9 weeks in LCR and HCR Sham or OVX rats (*n* = 8–10/group). ANOVA followed by Tukey post hoc analysis. Unlike letters are significantly different: *P* < 0.01.

**Figure 2:**
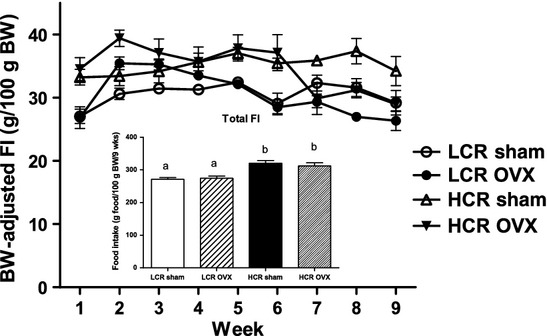
HCR rats consume more energy per gram of body weight than LCR irrespective of OVX. Weekly body weight adjusted food intake (line graph); total body weight-adjusted food intake (g food/100 g BW; inset) in LCR and HCR rats with and without OVX 9 *n* = 8–10/group). HCR rats consumed more food per gram body weight than LCR animals regardless of OVX. ANOVA followed by Tukey post hoc analysis. Unlike letters are significantly different; *P* < 0.01.

Baseline age, body weights, and best running distance data are listed in Table1. As per the characterization of the running phenotype, the best baseline running distance for the LCR group was significantly lower than that for the HCR animals (*P *<* *0.001). Running distance was not measured post-OVX. At baseline, HCR weighed significantly less than LCR (*P *<* *0.001), and in the Sham condition, remained so through study termination (*P *<* *0.01). With OVX, both the LCR and HCR gained significantly more weight than the respective Sham animals (Fig.1, *P* < 0.001), however, HCR OVX weighed significantly less than LCR OVX throughout the study. Body weight adjusted food intake (g/100 g BW) for the study period (9 weeks) was not different between OVX and Sham groups, but was significantly greater in HCR versus LCR overall (Fig.2, *P* < 0.01).

**Table 1:** Animal characteristics, tissue weights, and glucose measures

	LCR Sham	LCR OVX	HCR Sham	HCR OVX
Age at baseline (weeks)	40 ± 2.8	40.8 ± 2.6	41.5 ± 2.0	41.7 ± 2.1
Best running distance (baseline; m)	277 ± 56	238 ± 69	2133 ± 313*	2138 ± 212*
Baseline BW (g)	267 ± 13	267 ± 19	224 ± 21*	219 ± 13*
Termination BW (g)	267 ± 18	308 ± 16†	235 ± 22*	257 ± 16*†
Uterine weight (g)	1.1 ± 0.2	0.47 ± 0.1†	1.1 ± 0.2	0.53 ± .2†
Visceral AT (g)	2.9 ± 1.1	3.8 ± 1.2	1.8 ± 0.53	2.4 ± 1.2
Subcutaneous AT (g)	2.1 ± .64	3.1 ± 0.77†	1.0 ± 0.27*	1.5 ± 0.39
Perirenal AT (g)	2.3 ± 0.84	3.5 ± 1.0†	1.3 ± 0.45*	2.1 ± 0.53†
Ovarian AT (g)	4.7 ± 1.7	6.0 ± 1.8	3.1 ± 1.2	3.7 ± 1.7
GTT AUC	12868 ± 1537	11733 ± 1454	13360 ± 1764	12169 ± 1295
Fasting glucose (mmol/L)	7.5 + 1.4	7.1 ± 0.38	7.0 ± 0.72	6.02 ± 0.57

Values are mean ± SD.

GTT AUC = glucose area under the curve over 120 min following i.p. injection of 1.5 g/kg dextrose at week 7 following OVX.

* *P* = <0.05 HCR versus LCR.

† *P* = <0.05 Sham versus OVX. Group and treatment differences were analyzed via ANOVA with Tukey post hoc analysis.

Tissue weights are listed in Table1. Sham LCR rats had significantly more subcutaneous (SAT; *P* < 0.001) and perirenal (*P* = 0.041) adipose tissue (AT) than Sham HCR, but did not differ in visceral (VAT) or ovarian AT. Successful OVX was confirmed by a significant decrease in uterine weight in OVX (0.5 ± 0.15 g) versus sham (1.1 ± 0.018 g) animals (Table1, *P* < 001). Percent SAT and perirenal AT were significantly greater in LCR OVX relative to LCR Sham (*P* = 0.051 and *P* = 0.041, respectively). However, in HCR OVX versus Sham, only perirenal AT was significantly greater (*P* = 0.037). There were no significant OVX differences in ovarian or visceral AT depot weights.

#### Fasting glucose and glucose tolerance test

After a 4-h fast, the area under the curve (AUC) response to an i.p. glucose load (ipGTT) did not differ between groups or treatments (Table1).

#### Liver triglycerides

**Figure 3:**
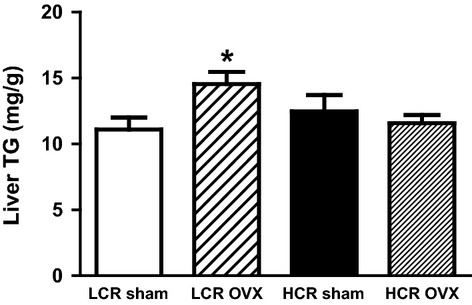
HCR rats are protected from liver triglyceride accumulation with OVX. Liver TG (mg/g tissue) was significantly higher in LCR OVX relative to LCR Sham, with no difference between HCR OVX and HCR Sham (*n* = 7–10/group). *P = 0.05.

As depicted in Figure3, OVX led to a significant increase in liver triglycerides (TG) in the LCR group (*P* = 0.049), but not in the HCR animals (*P* = 1.0).

#### Bacterial richness and Firmicutes:Bacteroidetes ratio are differentially altered with OVX

Potential differences in fecal microbiota between treatment groups were investigated via multiple measures. These included richness (the total number of OTU’s detected in a sample), evenness (a measure of OTU distribution), and diversity (which accounts for OTU number and distribution). Overall, no differences were observed in diversity or evenness across groups, but LCR rats showed significantly higher fecal bacterial richness (number of observed species) in OVX versus all other groups. The Chao1 richness estimate, which uses bootstrapping techniques to estimate species potentially not detected due to incomplete sampling, was not significantly different across groups (Table2).

**Table 2:** Bacterial diversity

Group	sobs	chao	shannon	simpson	invsimpson
hcr_ovx1	120	165.0417	3.3252	0.094954	10.5315
hcr_ovx2	135	207.5455	3.7956	0.047339	21.124231
hcr_ovx3	121	181.2727	3.3054	0.098634	10.1385
hcr_ovx4	116	222.4	3.2694	0.08866	11.279
hcr_sham1	127	257.7143	3.3829	0.083257	12.011
hcr_sham2	131	213.5	3.7414	0.05273	18.9644
hcr_sham3	119	178.3684	3.5367	0.062653	15.961
hcr_sham4	117	223.0714	3.273	0.090163	11.0911
lcr_ovx1	126	186.06	3.64	0.07	15.18
lcr_ovx2	136	211.12	3.48	0.08	13.2
lcr_ovx3	151	227.25	4.17	0.03	37.21
lcr_ovx4	123	168.32	3.59	0.06	16.41
lcr_sham1	111	179.9	3.08	0.12	8.37
lcr_sham2	117	192	3.49	0.07	14.39
lcr_sham3	104	189	2.72	0.17	5.75
lcr_sham4	115	170.65	3.35	0.1	10.46
P value*	0.02536	0.4455	0.1472	0.1022	0.1004

Sobs, the observed richness (number of species); chao, the Chao1 estimator of actual richness; shannon, Shannon Index (measure of diversity); simpson, Simpson Index (measure of diversity); invsimpson, Inverse Simpson Index (measure of evenness).

* Via the nonparametric Kruskal–Wallis test.

**Figure 4:**
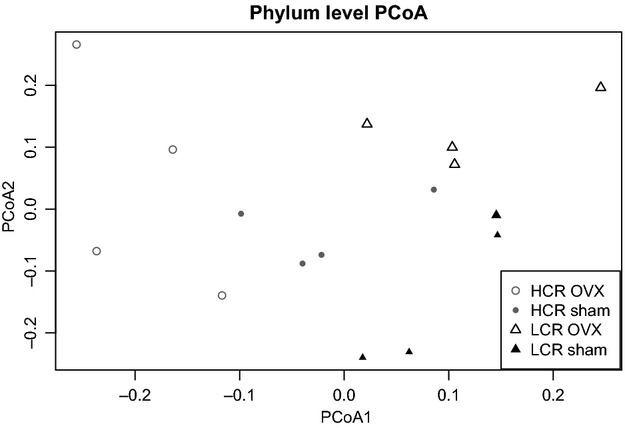
Principal coordinate analysis (PCoA) illustrates clustering of fecal bacterial community by OVX status with LCR rats and HCR rats. PCoA utilized the Jaccard distance matrix with OTU relative abundances at 3% genetic distance. Cluster centroids are significantly different for LCR OVX versus LCR Sham and LCR OVX versus HCR OVX, but not for HCR OVX versus HCR Sham or LCR Sham versus HCR Sham via AMOVA test; *P* < 0.05.

Community composition of the gut microbiota was compared using the traditional Jaccard index, which considers both total and shared OTUs. Significant differences in the four groups were first identified via the multivariate adonis test (*P* < 0.01), and further investigated using molecular analysis of variance (AMOVA). The AMOVA test revealed significant differences between the fecal bacterial communities of OVX and Sham LCR rats (*P* = 0.02) and LCR OVX versus HCR OVX rats (*P* = 0.01). No significant difference in fecal bacterial communities was observed in LCR versus HCR Sham rats (*P* > 0.05) via AMOVA. The PCoA visualizing the Jaccard distance matrix confirmed differences in composition of fecal microbiota in clear separation of the four groups (Fig.4).

**Figure 5:**
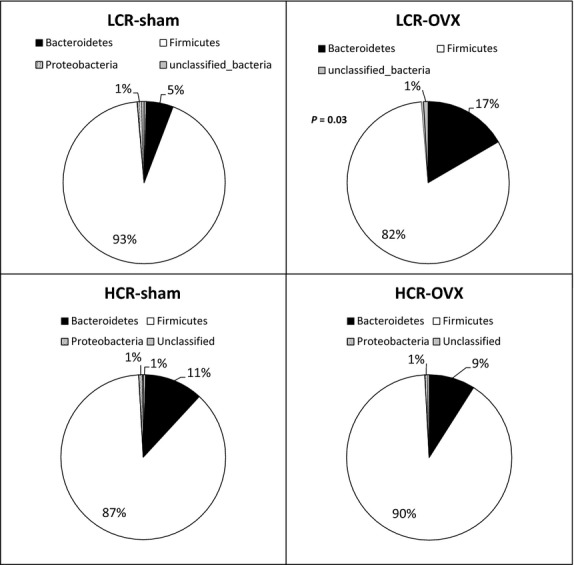
OVX is associated with decreased *Firmicutes:Bacteroidetes* ratio in LCR, but not HCR rats. Phyla-level bacterial composition of fecal samples was determined for HCR and LCR rats both with and without OVX (*n* = 4/group). *Firmicutes* were increased and *Bacteroidetes* were decreased in LCR OVX relative to LCR Sham; *P* = 0.021. No difference in bacterial phyla composition was observed in HCR OVX compared to HCR Sham.

The metastats test identified 29 OTUs with significantly different abundance between the four groups (Table2). In Sham LCR and HCR rats, two OTUs had higher abundance in LCR versus HCR rats from unclassified genera of *Clostridiales* and *Ruminococcaceae* (Table2A). Nine OTUs had increased abundance in Sham HCR versus LCR rats, including SCFA-producing *Bifidobacterium* spp. (Table3A). When comparing OVX versus Sham conditions, while OTU’s from *Bacteroides*, *Barnesiella*, and *Prevotella* were significantly more abundant in LCR OVX versus LCR Sham rats, the abundance of OTUs from these three genera were significantly lower in HCR OVX versus HCR Sham (Table3B and C). Average fecal microbial composition on a phyla level was also examined for the four groups. Overall, LCR OVX rats showed increased abundance of *Bacteroidetes* and decreased abundance of *Firmicutes* which resulted in a significantly lower *Firmicutes:Bacteroidetes* ratio (Fig.5, Table4A) when compared to LCR Sham rats. In contrast, there was no difference in *Firmicutes:Bacteroidetes* ratio between HCR OVX and HCR Sham (Fig.5, Table4B). In summary, both HCR and LCR rats show altered composition of fecal microbial communities with OVX versus Sham, with LCR rats exhibiting both increased species richness and a greater number of increasing taxa compared to HCR rats.

**Table 3:** Taxonomy table

Name	Direction	*P*-value	Consensus taxonomy
(A) LCR Sham versus HCR Sham			
Otu004	Lower	0.0017	*Bacteroides* spp.
Otu041	Lower	0.0325	Unclassified bacteria
Otu046	Lower	0.0190	*Alistipes* spp.
Otu059	Lower	0.0216	*Sporobacter* spp.
Otu077	Lower	0.0484	*Bacteroides* spp.
Otu091	Lower	0.0103	Unclassified Lachnospiraceae
Otu095	Lower	0.0357	Unclassified Firmicutes
Otu096	Higher	0.0231	Unclassified Clostridiales
Otu099	Lower	0.0370	*Bifidobacterium* spp.
Otu119	Lower	0.0336	Unclassified Clostridiales
Otu209	Higher	0.0289	Unclassified Ruminococcaceae
(B) LCR OVX versus Sham			
Otu001	Lower	0.0194	*Moryella* spp.
Otu011	Lower	0.0376	*Sporobacter* spp.
Otu014	Higher	0.0313	*Oscillobacter* spp.
Otu033	Higher	0.0418	Unclassified *Ruminococcaceae* spp.
Otu063	Higher	0.0058	*Barnesiella* spp.
Otu085	Higher	0.0141	Unclassified Ruminococcaceae spp.
Otu088	Higher	0.0435	Unclassified Porphyromonadaceae spp.
Otu091	Higher	0.0014	*Anaerosinus* spp.
Otu095	Higher	0.0007	Unclassified Lachnospiraceae spp.
Otu113	Higher	0.0007	*Barnesiella* spp.
Otu119	Higher	0.0420	*Prevotella* spp.
Otu209	Lower	0.0272	*Eggerthella* spp.
Otu213	Higher	0.0272	*Barnesiella* spp.
Otu276	Higher	0.0272	*Bacteroides* spp.
(C) HCR OVX versus Sham			
Otu006	Higher	0.0435	Unclassified Lachnospiraceae spp.
Otu024	Higher	0.0422	*Abiotrophia* spp.
Otu028	Lower	0.0202	*Bacteroides* spp.
Otu068	Higher	0.0136	*Subdoligranulum* spp.
Otu089	Lower	0.0321	*Barnesiella* spp.
Otu104	Lower	0.0361	*Acetanaerobacterium* spp.
Otu119	Lower	0.0136	*Prevotella* spp.
Otu121	Lower	0.0257	*Acetanaerobacterium* spp.
Otu156	Lower	0.0257	*Barnesiella* spp.
(D) LCR OVX versus HCR OVX			
Otu001	Lower	0.0209	*Moryella* spp.
Otu005	Lower	0.0028	*Sporobacter* spp.
Otu008	Lower	0.0014	*Sporacetigenium* spp.
Otu016	Higher	0.0103	Unclassified Rumincoccaceae
Otu026	Lower	0.0008	Unclassified Lachnospiraceae
Otu028	Higher	0.0052	*Bacteroides* spp.
Otu039	Higher	0.0036	*Ruminococcus* spp.
Otu040	Higher	0.0026	Unclassified Lachnospiraceae
Otu042	Lower	0.0006	Unclassified Lachnospiraceae
Otu045	Higher	0.0023	*Paraprevotella* spp.
Otu047	Higher	0.0026	*Barnesiella* spp.
Otu056	Higher	0.0022	*Barnesiella* spp.
Otu063	Higher	0.001	*Barnesiella* spp.
Otu066	Higher	0.0009	*Prevotella* spp.
Otu068	Lower	0.0003	*Subdoligranulum* spp.
Otu079	Lower	0.0002	*Sporobacter* spp.
Otu113	Higher	0.0003	*Barnesiella* spp.
Otu119	Higher	0.0006	*Prevotella* spp.
Otu213	Higher	0.0004	*Barnesiella* spp.
Otu276	Higher	0.0004	*Bacteroides* spp.

Shaded text highlights changes in similar bacteria between groups.

**Table 4:** Percent relative abundance of fecal bacterial phyla

	Actinobacteria	Bacteroidetes	Firmicutes	Proteobacteria	Unclassified	F:B ratio
(A) LCR						
lcr_ovx1	0%	16%	82%	0%	1%	4.986
lcr_ovx2	0%	17%	82%	1%	0%	4.909
lcr_ovx3	0%	28%	71%	1%	0%	2.536
lcr_ovx4	0%	28%	71%	1%	0%	2.533
lcr_sham1	0%	5%	93%	1%	0%	17.366
lcr_sham2	0%	12%	83%	1%	3%	6.992
lcr_sham3	0%	12%	87%	0%	0%	7.189
lcr_sham4	0%	12%	84%	3%	2%	7.242
average	0%	16%	82%	1%	1%	6.719
P value*	0.237	0.021	0.021	0.564	0.248	0.021
(B) HCR						
hcr_ovx	0%	9%	90%	1%	0%	10.080
hcr_ovx	0%	14%	85%	0%	0%	5.937
hcr_ovx	0%	5%	92%	2%	0%	17.220
hcr_ovx	1%	7%	90%	3%	0%	13.706
hcr_sham	0%	11%	87%	1%	0%	7.635
hcr_sham	0%	16%	83%	1%	0%	5.178
hcr_sham	0%	8%	91%	1%	0%	11.351
hcr_sham	0%	5%	94%	0%	0%	18.636
average	0%	9%	89%	1%	0%	9.420
P value*	0.149	0.773	1.000	0.773	0.149	0.773

* Via the nonparametric Kruskal–Wallis test.

### Gut permeability is not significantly changed with bacterial changes based on group or treatment

Gut permeability was measured at week 5 via oral gavage of FITC-dextran in a subset of rats (*n* = 4) from each group (data not shown). Gut permeability did not reach statistical significance with Tukey post hoc analysis (*P* = 0.41). There was also no significant difference in the HCR OVX versus Sham groups (*P* = 1.0). To further explore gut integrity, plasma endotoxin was measured at study termination, however, endotoxin was undetectable in all of our samples (data not shown).

### SCFA are greater in HCR than LCR Sham, and are decreased in both groups with OVX

**Figure 6:**
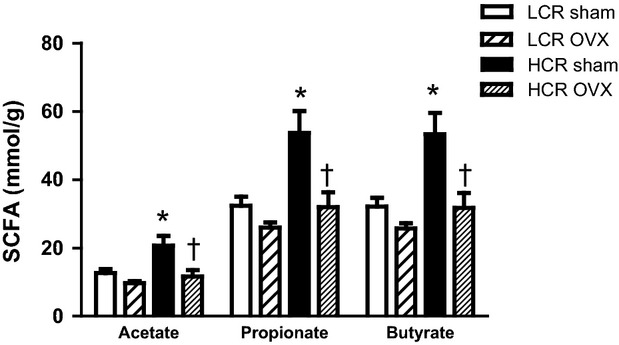
Fecal short-chain fatty acids (SCFA) are significantly higher in HCR Sham relative to LCR Sham and are decreased in both groups with OVX. Fecal SCFA were analyzed via gas chromatography (*n* = 4/HCR Sham, 5/HCR OVX, 9/LCR Sham, 10/LCR OVX). *P ≤ 0.01 HCR versus LCR, †*P* ≤ 0.01 HCR Sham versus HCR OVX.

Short-chain fatty acids (acetate, propionate, butyrate) were significantly higher in HCR Sham versus LCR Sham (Fig.6; *P* = 0.004, acetate; *P* = 0.001, propionate and butyrate). With OVX, however, all SCFA significantly decreased in the HCR group (*P* = 0.015, acetate; *P* = 0.010 propionate and butyrate), but were unchanged in the LCR group (*P* > 0.30 for all SCFA).

## Discussion

We set out to determine if innate aerobic capacity is protective against the metabolic implications of ovariectomy (OVX) in rats and if these factors are associated with gut microflora diversity and overall gut health. We demonstrated that rats with relatively greater intrinsic aerobic capacity were modestly protected against increased adipose tissue and liver triglyceride deposition in response to OVX compared to those with lower aerobic capacity. Moreover, these differences coincided with altered gut ecology and fecal short-chain fatty acids (SCFAs). To the best of our knowledge, this is the first report of the effects of OVX and gut microbe profiling in the LCR/HCR model.

Although there were no differences in total or percent body weight gain with OVX between groups, we observed modest protection against OVX-induced adiposity in the HCR relative to LCR rats. While this manuscript was in process, Vieira-Potter et al. published similar findings that HCR rats are modestly protected against OVX-associated body weight gain, adiposity, and insulin resistance (Vieira-Potter et al. 2015). Adipose tissue weight varied based on depot in response to OVX in LCR versus HCR animals, but was overall greater in LCR OVX.

We did not measure physical activity, but since food intake was not different between OVX and Sham in either group, and previous rodent studies have reported decreased spontaneous physical activity with OVX (Gorzek et al. 2007; Rogers et al. 2009), it is likely that the OVX-induced body and tissue weight increases were partly due to decreased physical activity in both LCR and HCR. Indeed, Vieira-Potter et al. reported that spontaneous physical activity and total energy expenditure were both significantly decreased in the OVX condition, regardless of group (Vieira-Potter et al. 2015). Given that exercise training in OVX rats results in protection from BW and AT gain (Pighon et al. 2011), our results, and those of Vieria-Potter et al. indicate that innate aerobic capacity per se cannot overcome the OVX-induced decrease in spontaneous physical activity and energy expenditure. Contrary to Vieria-Potter, we did not observe a decrease in food intake with OVX in either LCR or HCR animals.

The elevation in liver TG in LCR OVX relative to LCR Sham is consistent with previous reports of OVX in rats (Pighon et al. 2011). That we did not see equivalent elevation in HCR OVX relative to HCR Sham, further suggests some level of metabolic protection. In the current study, there were no significant group or treatment differences in fasted glucose or ipGTT AUC, despite differences in body weight and adiposity. Previous studies report mixed results, with varying degrees of glucose and insulin sensitivity (Johnsen et al. 2013; Choi et al. 2014; Vieira-Potter et al. 2015). This may be due to differences in age, generation of the animals, diet composition, housing environment, or experimental technique.

Much attention has recently been paid to the composition of the gut microbiota with respect to metabolic health. Given that others have shown the ability for exercise to alter gut ecology (Matsumoto et al. 2008; Choi et al. 2013), we hypothesized that the relative aerobic capacity of the LCR/HCR rats would confer different populations of gut microbes. While our observed *Firmicutes:Bacteroidetes* ratio in LCR (6.7) versus HCR (9.4) was consistent with the reported association of a decreased *Firmicutes:Bacteroidetes* ratio with a lean phenotype (Ley 2010) and prescribed exercise (Choi et al. 2013), there was no significant difference in overall bacterial community structure between LCR and HCR Sham animals. However, SCFA concentrations were significantly higher in HCR Sham relative to LRC Sham animals, suggesting *functional* differences between the microbiomes of these animals that are not captured by taxonomy-based comparisons.

There is a paucity of literature regarding changes in gut microbiota with loss of estrogen, but limited human data indicate that gut ecology may change under these circumstances, accompanied by metabolic consequences (Flores et al. 2012; Fuhrman et al. 2014). In the current study, OVX resulted in greater richness of fecal microbiota in LCR, but not HCR rats. This is reflected at the phyla level by an increase in the Bacteroidetes, which contain commensals that degrade polysaccharides, and Gram-negative species that produce proinflammatory endotoxins. Specific taxa increases in LCR with OVX included unclassified genus including *Barnesiella, Bacteroides,* and *Prevotella*, the latter two of which are known pathogens (Sack et al. 1992; Koeth et al. 2013). Endotoxin, or lipopolysaccharide (LPS), is a component of the Gram-negative bacteria cell wall, and can increase with increased Gram-negative bacterial population to levels that elicit an inflammatory immune response (Heumann and Roger 2002). Interestingly, these same species were significantly lower in HCR OVX group relative to HCR Sham, suggesting that aerobic capacity may be protective against enteric pathogen invasion. These divergent results may be directly due to differences in inherent aerobic capacity, or to the various other differences identified in this model, including nonresting energy expenditure (Gavini et al. 2014), age-dependent cardiac remodeling (Ritchie et al. 2013), intrinsic mitochondrial capacity (Seifert et al. 2012), or endocrine stress responsiveness (Waters et al. 2010).

A greater relative level of pathogenic bacteria, as seen in the LCR OVX relative to LCR Sham is associated with intestinal permeability and systemic inflammation (Tremaroli and Backhed 2012). At week 5, we did not observe changes in gut permeability as measured by the appearance of FITC-dextran in systemic circulation. This measure was done mid-way through the study to minimize perturbations to final outcomes; we cannot say how this might have changed over the subsequent 4 weeks. To further characterize possible metabolic consequences of a disrupted microbial community with OVX, we measured plasma endotoxin at termination. Multiple studies have measured endotoxin with a similar *Limulus* Amebocyte Lysate (LAL) method used in the current study (Cani et al. 2008; Kim et al. 2012; Woting et al. 2014), however, we could not detect endotoxin in any of our samples (data not shown). This may be due to the short duration of our study (9 weeks) that our rats were on a normal chow diet (a single high-fat meal can raise plasma endotoxin within 4 h in normal weight human subjects) (Harte et al. 2012), or that the method is not appropriate for detection of serum endotoxin.

The most robust outcome of the current study was the considerable difference in fecal short-chain fatty acids (SCFA; acetate, propionate, butyrate) in LCR versus HCR. SCFA are produced by the metabolism of fiber by gut microbes and have been identified as a host energy source, as peripheral and central signaling molecules regulating food intake and energy metabolism through receptors in the brain, adipose tissue, and immune cells (reviewed in [Kuwahara 2014]), and as regulators of fatty acid metabolism (Ge et al. 2008). In humans, total SCFA have been positively correlated with F:B ratio, and negatively correlated with *Bacteroidetes* (Fernandes et al. 2014). In the current study, HCR rats had significantly more fecal SCFA than LCR rats in the Sham condition, consistent with increased SCFA with exercise training (Matsumoto et al. 2008), and corresponding to significantly higher *Bifidobacterium* (Gram-positive; prolific SCFA producers (Gibson and Roberfroid 1995)),in HCR Sham than LCR Sham. OVX resulted in significantly reduced SCFA levels in HCR to the level seen in LCR Sham, and nonsignificantly in LCR. This indicates that the OVX-mediated changes in SCFA metabolism may be independent of innate aerobic capacity. However, the HCR maintained a higher total level of SCFA than LCR, which may play a role in the modest protection from adiposity and weight gain observed in the HCR. This assertion is consistent with our liver TG data in that previous studies have shown that conventionally raised rats have higher liver TG than germ-free animals (Velagapudi et al. 2010), and that overexpression of the SCFA receptor, GPR43 results in protection from high-fat diet induced hepatic TG accumulation (Kimura et al. 2013).

A weakness of this study is that we were unable to measure physical activity. The HCR rats have been shown to have increased spontaneous activity relative to LCR rats (Novak et al. 2010), and since decline in physical activity plays a major role in weight gain after menopause, this would be a critical piece of data. Fortunately, Vieria-Potter et al. confirmed that physical activity is significantly decreased in both LCR and HCR with OVX (Vieira-Potter et al. 2015). Measuring SCFA in the cecum would also benefit the robustness of this measure, as the fecal SCFA are not a direct measure of production or utilization. Although OVX is a well-accepted model of menopause, there are several major differences between the procedure and natural menopause that must be considered.

The strengths of the study include the LCR/HCR model itself, the age of the animals, and the diet. The polygenic nature of the LCR/HCR rats may be more representative of the human population than an inbred strain. Moreover, this model allowed us to study aerobic capacity without confounding of the most recent bout of exercise. Similarly, that these animals were not ovariectomized until they were mature adults at 32 weeks, may be more physiologically similar to natural menopause, as opposed to the more standard 12–16 weeks, when they may still be growing. We also employed a purified, phytoestrogen-free chow diet. Given that dietary phytoestrogens can alter gut ecology and other metabolic parameters, this diet allowed us to examine the effects of OVX without dietary interference. This is the first study to examine differences in gut microbiota in the LCR/HCR model at baseline and with loss of endogenous estrogen.

In summary, consistent with studies in exercised rats, and results recently published in LCR/HCR rats (Vieira-Potter et al. 2015), HCR rats are moderately protected from some of the early metabolic consequences of endogenous estrogen removal via OVX. Protection of the liver from accumulating lipid is particularly important in maintaining glucose homeostasis in the long term. The inherent differences in gut ecology between the LCR and HCR animals in the Sham condition, and the inverse changes in the groups with OVX suggest a link between estrogen metabolism and gut microbe maintenance. The decrease in SCFA in both groups with OVX is also consistent with estrogen regulation of energy metabolism and gut ecology. However, we are unable to separate influences in fecal microbiota due to running capacity or innate aerobic capacity from other potential differences between LCR and HCR rats such as those mentioned above, or are yet undiscovered. Overall, the current data support a protective role of the HCR phenotype against OVX-associated metabolic assault, and propose a novel microbial-associated mechanism for estrogen-mediated control of energy metabolism. Further studies will need to focus on expanding the functional characterization of the microbial community with OVX.
